# Supplementary material for: Predicting visual function by interpreting a neuronal wiring diagram
Source: Nature. 2024 Oct 2;634(8032):113–23. doi: 10.1038/s41586-024-07953-5 (PMC11446822; doi:10.1038/s41586-024-07953-5)
Supplement: Supplementary file 6 — CRF and ERF predictions for individual TmY4 and TmY9 cells. Analogous to Supplementary Data 3, but for TmY target types. Shown are the top four monosynaptic pathways, the strongest pathway passing through each of the top ten intermediary types (ranking from Extended Data Fig. 7), and the trisynaptic pathway Tm1–TmY–Dm3–TmY (see the section entitled Prediction of spatial normalization). [file 41586_2024_7953_MOESM6_ESM.zip › DataS4/TmY9q/720575940614212674.pdf]

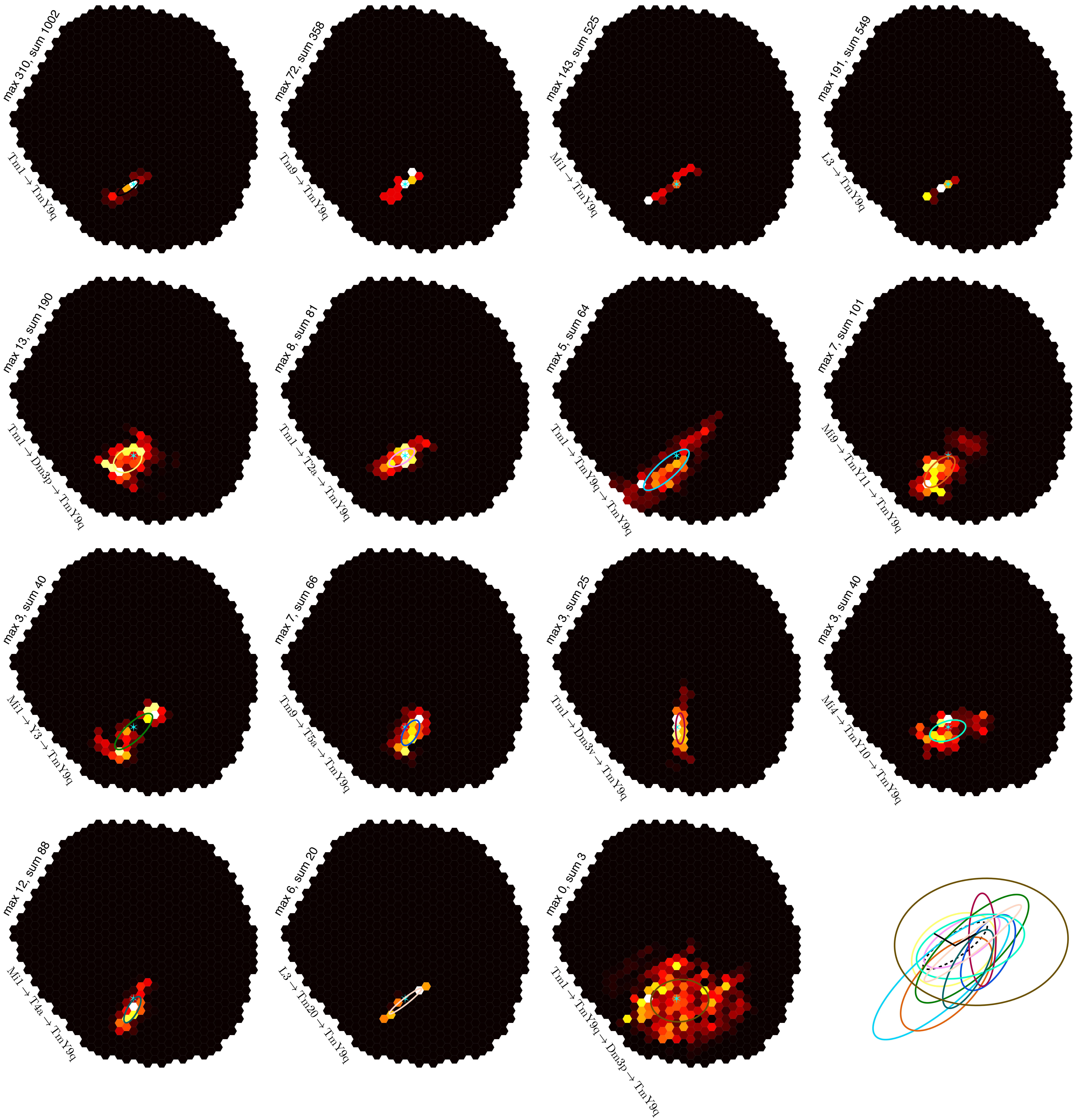
max 310, sum 1002
Tm1 → TmY9q
max 72, sum 358
Tm9 → TmY9q
max 143, sum 525
Mi1 → TmY9q
max 191, sum 549
L3 → TmY9q
max 13, sum 190
Tm1 → Dm3p → TmY9q
max 8, sum 81
Tm1 → T2a → TmY9q
max 5, sum 64
Tm1 → TmY9q → TmY9q
max 7, sum 101
Mi9 → TmY11 → TmY9q
max 3, sum 40
Mi1 → Y3 → TmY9q
max 7, sum 66
Tm9 → T5a → TmY9q
max 3, sum 25
Tm1 → Dm3v → TmY9q
max 3, sum 40
Mi4 → TmY10 → TmY9q
max 12, sum 88
Mi1 → T4a → TmY9q
max 6, sum 20
L3 → Tm20 → TmY9q
max 0, sum 3
Tm1 → TmY9q → Dm3p → TmY9q
